# Perinatal Mental Health during COVID-19 Pandemic: An Integrative Review and Implications for Clinical Practice

**DOI:** 10.3390/jcm10112406

**Published:** 2021-05-29

**Authors:** Julia Suwalska, Maria Napierała, Paweł Bogdański, Dorota Łojko, Katarzyna Wszołek, Sara Suchowiak, Aleksandra Suwalska

**Affiliations:** 1 Department of Treatment of Obesity, Metabolic Disorders and Clinical Dietetics, Poznan University of Medical Sciences, 60-569 Poznan, Poland; pbogdanski@ump.edu.pl; 2 Department of Mental Health, Chair of Psychiatry, Poznan University of Medical Sciences, 60-572 Poznan, Poland; mnapierala@ump.edu.pl (M.N.); lojko@ump.edu.pl (D.Ł.); sara.suchowiak@o2.pl (S.S.); asuwalska@ump.edu.pl (A.S.); 3 Department of Mother and Child Health, Poznan University of Medical Sciences, 60-535 Poznan, Poland; katarzyna.wszolek@ump.edu.pl

**Keywords:** COVID-19, pandemic, perinatal mental health, depression, anxiety, restrictions, social support, telemedicine, integrative review

## Abstract

The COVID-19 pandemic and measures implemented to decelerate its spread have consequences for mental health of societies. The aim of our review was to analyze depressive and anxiety symptoms in perinatal women. The search used PubMed and Web of Science databases. Most studies showed an increase in the prevalence of depression and/or anxiety symptoms. Risk factors identified in our study were mainly related to the possibility of COVID-19 infection, changes in the organization of perinatal care, social isolation and financial problems. Protective factors included social support, the woman’s own activity and knowledge about COVID-19. The results of our study point to the importance of the mental health screening including suicide risk assessment in perinatal women. Much of the mental health needs of perinatal women can be met in primary or perinatal care services; however, women with mental health issues should be offered psychiatric consultations and psychological support, and sometimes urgent psychiatric hospitalization is necessary. Healthcare professionals should provide information addressing uncertainty about COVID-19, organization of midwifery and medical care as well as mental health problems and how to get help. Mental health interventions in pregnant women may involve planning physical activity and encouraging to engage in online social activities.

## 1. Introduction

On 31 December 2019, China reported a cluster of cases of pneumonia in Wuhan, Hubei Province [1], associated with a novel coronavirus [2]. In February 2020, the World Health Organization (WHO) named the disease caused by it as COVID-19 [3]. Soon, COVID-19 became a threat to global health [4] and spread to almost all countries worldwide and evolved into a pandemic [5]. A year after the original outbreak—on 31 December 2020, the total cumulative count (total cases worldwide) of coronavirus cases including deaths and recovered or discharged patients (cases with an outcome) stood at 83,969,640, and novel coronavirus daily cases worldwide stood at 759,004 [6].

Governments around the world have responded to the coronavirus disease 2019 (COVID-19) pandemic with unprecedented policies aimed to decrease virus transmission by reducing contact among individuals within or between populations. Many policies, such as closing schools and restricting populations to their homes, impose large and visible costs on society [7]. Mental health consequences of COVID-19 pandemic in the general population are associated with quarantine and isolation as well as worrying about one’s own health, family, friends and acquaintances being infected [8,9]. Public health measures implemented to decelerate the spread of COVID-19, such as physical distancing and travel restrictions, might help to alleviate the burden of healthcare systems, but have unintended consequences for women and families [10]. Such consequences could include gender-based and family violence, as well as a reduction in preventive healthcare-seeking behaviors, such as prenatal care and well-child visits [9,10]. Pregnant women and new mothers are a particularly vulnerable group. In this population, an increase in depression prevalence and the exacerbation of other mental health concerns has been observed [9,10]. It should be stressed that antenatal and postpartum depression may pose an additional threat to the woman’s life since perinatal suicidality is nowadays considered one of the leading causes of maternal mortality in the first 12 months postpartum [11]. Prenatal depression and anxiety also have negative effects on fetal growth and neonatal outcome including a greater incidence of prematurity and low birthweight [12]; symptoms of depression are associated with behavioral or mood disturbances in offspring that can increase their risk of depression in adolescence and adulthood. [13]. Efforts to reduce the prevalence of depression and anxiety should be a public health priority [14].

Researchers from many countries have undertaken studies to determine the impact of the COVID-19 pandemic on depressive and anxiety symptoms in pregnant women and new mothers. A few systematic reviews with meta-analysis following the Preferred Reporting Items for Systematic Reviews and Meta-Analyses (PRISMA) guidelines [15] have been carried out. The results of the reviews concerning antenatal psychological symptoms indicate increased severity of anxiety [16] and increased prevalence of anxiety and depression [17,18,19] in pregnant women during the COVID-19 pandemic. Based on a systematic review with meta-analysis, Yan et al. [20] found high rates of anxiety, depression and insomnia in pregnant women and new mothers. No comprehensive analysis of factors associated with the worsening of mental state of pregnant and postpartum women during COVID-19 pandemic has been reported.

The aim of the current study was to analyze the prevalence and intensity of mental health problems in pregnant women and new mothers during the COVID-19 pandemic, examine risk factors of depression and anxiety, as well as protective factors, and consider the application of the findings in perinatal care.

## 2. Materials and Methods

Integrative review methods were used as they allow for consideration of studies with varying methodologies and rigor [21].

### 2.1. Search Strategy and Study Selection

We searched the PubMed and Web of Science databases from inception until 4 November 2020. The search terms were: (COVID-19 OR SARS-CoV-2 OR coronavirus) AND (depression OR anxiety OR stress OR mood) AND (pregnancy OR pregnant OR postpartum OR perinatal). The literature search was performed independently and in duplicate by two authors (J.S. and M.N.). The titles and abstracts of the retrieved articles were screened to exclude records that did not meet the inclusion criteria. J.S. and M.N. further reviewed full-text articles. Disagreements between reviewers regarding the study selection were resolved by consensus or by the decision of a third independent reviewer (A.S.). A manual search of references of pertinent articles was performed to identify additional relevant studies.

### 2.2. Eligibility Criteria

Studies were eligible for inclusion if they (1) assessed the mental health status of pregnant women or new mothers during the first wave of the COVID-19 pandemic, (2) used standardized and validated scales for measurement of depressive and anxiety symptoms and (3) were written in English. Only original articles were included. If publications presented data from the same study, only the publication with the largest sample size was included.

### 2.3. Data Collection Process

K.W. and S.S. performed the data extraction independently using an Excel spreadsheet. The data were obtained from tables or figures if no direct information was available in the text. Disagreements regarding the data extraction were resolved by consensus or by the decision of A.S.

### 2.4. Data Items

The data items included the following: research type, study title, first author, publication year, country, time of the study, sample size and characteristics of participants, methods of recruitment, screening tools and study outcomes (prevalence of depressive and/or anxiety symptoms, risk and protective factors). No assumptions and simplifications were made.

### 2.5. Quality Assessment

J.S. and K.W. independently evaluated the risk of bias of the included studies using a modified form of the Newcastle–Ottawa scale [22,23]. Disagreements regarding quality assessments were resolved by A.S. Quality assessment criteria were the following: sample representativeness and size, comparability between respondents and non-respondents, ascertainment of depression and anxiety and adequacy of descriptive statistics. The total quality score ranged between 0 and 5. Studies scoring ≥ 3 points were regarded as low risk of bias, compared to the studies assessed with <3 points that were regarded as high risk of bias [22].

## 3. Results

### 3.1. Search Results

**Figure 1:**
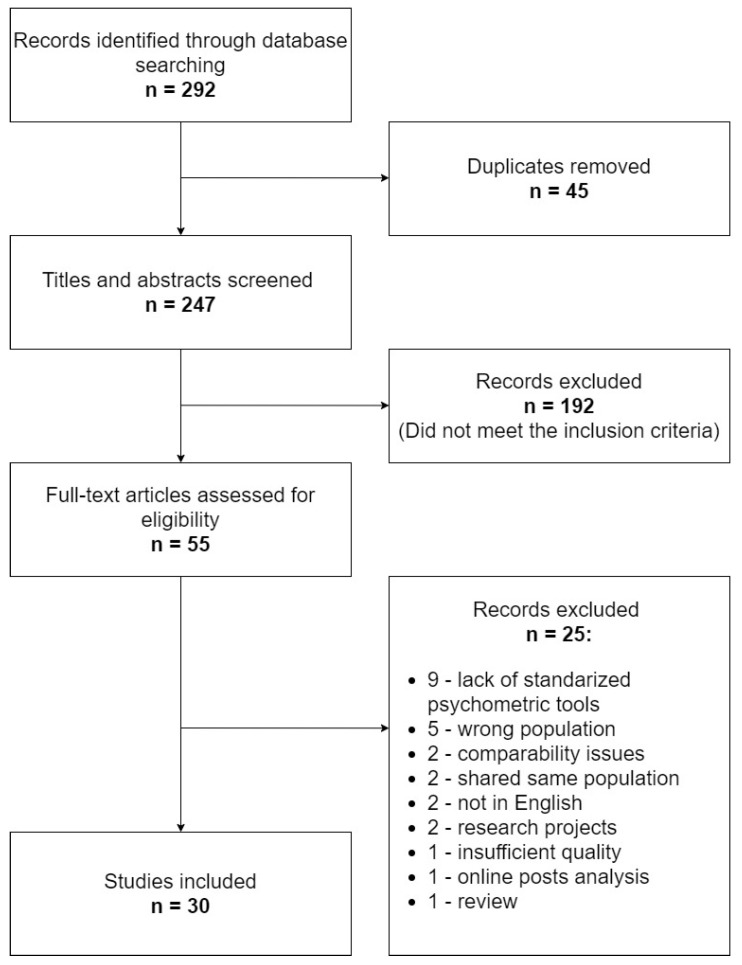
Search flowchart for the selection of articles.

Our initial search identified 292 items. Following removal of 45 duplicates, the titles and abstracts of the remaining 247 records were screened for relevance. Overall, 192 articles were excluded on the following grounds: wrong population (non-perinatal women, pregnant women infected with COVID-19, medical professionals, non-human studies), wrong topic (pharmacotherapy, other viral infections, breastfeeding, somatic diseases in pregnancy, etc.) and non-original articles. In total, 30 articles were included after full-text review. The reasons for the removal of 25 papers were as follows: lack of standardized psychometric tools, disparate populations studied, same populations studied, insufficient quality and non-English language, as well as being a research project, a review and an online posts analysis. Bibliographies of selected articles were screened to identify additional studies, which did not bring additional data. Figure 1 shows the article selection process.

### 3.2. General Characteristics of the Studies

Overall, 30 articles meeting the inclusion criteria were selected for analysis. The general characteristics of the selected studies are presented in Table 1. Studies were divided into those that assessed both depression and anxiety symptoms and those that assessed only depression or anxiety symptoms.

**Table 1:** Characteristics of the selected studies.

Ref	Country	Time	Participants	Recruitment	Tools
**Assessment of depression and anxiety**					
[24]	Belgium	No information	5866:2421 pregnant and 3445 breastfeeding women	Online survey	EPDS ^1^ GAD-7
[25]	Canada	04.2020	1754 pregnant women: 496 before and 1258 during the pandemic	Online survey—social media, advertisements in prenatal clinics	K10
[26,27]	Canada	04.2020	1987 pregnant women	Online survey—social media	EPDS ^1^ PROMIS PRAQ
[28]	China	02.2020	156 pregnant women	Online survey—social media and distributed by doctors	SDS SAS
[29]	China	02–03.2020	859:544 pregnant and 315 non-pregnant women	Online survey—social media	PHQ-9 GAD-7
[30]	China	03–06.2020 (remission phase)	625:516 pregnant and 109 postpartum women	Written survey—hospital patients	EPDS ^1^ GAD-7
[31]	Italy	03–05.2020	575:389 pregnant and 186 postpartum women	Online survey—social media	EPDS ^1^ STAI
[32]	Turkey	No information	260 pregnant women	Online survey—hospital patients	EPDS ^2^ BDI BAI
[33]	Turkey	04–05.2020	63 pregnant women before and during the pandemic	Face-to-face interviews—hospital patients	IDAS II BAI
[34]	Turkey	06–07.2020	403 pregnant women	Online survey—social media	HADS
[35]	USA	03–04.2020	31 pregnant and postpartum women	Phone interview and online survey—social media	PHQ-2 GAD-7
[36]	USA	04–05.2020	913 pregnant women	Online survey—medical records system	PHQ-2 GAD-7
[37]	Qatar	06–07.2020	288 pregnant and postpartum women	Written survey—hospital patients	PHQ-9 GAD-7
**Assessment of depression**					
[38]	China	01–02.2020	4124 pregnant women: 2839 before and 1285 after the epidemic declaration in China	Written survey—hospital patients	EPDS ^3^
[39]	Hong Kong	01–04.2020	4531 postpartum women (1 day and 1 week after delivery): 3577 before and 954 during the pandemic	Written survey—hospital patients	EPDS ^3^
[40]	Israel	03–05.2020	369 high-risk pregnant women: 279 before and 90 during the pandemic	Written survey—patients of high-risk pregnancy units	EPDS ^3^
[41]	Italy	03–05.2020	192 postpartum women: 101 before and 91 during the pandemic	Written survey—hospital patients	EPDS ^1^
[42]	Japan	05–06.2020	1777 pregnant women	Online survey—users of applications	EPDS ^1^
[43]	Turkey	06.2020	223 postpartum women (48 h after delivery)	Written survey—hospital patients	EPDS ^1^
[44]	USA	02–06.2020	485 pregnant women	Written survey—hospital patients	EPDS ^4^
[45]	USA	04.2020	2099 pregnant women	Online survey—social media	EPDS ^5^
**Assessment of anxiety**					
[46]	China	02.2020	1947 pregnant women	Online survey—social media and written survey—hospital patients	SAS
[47]	China	02.2020	308 pregnant women	Online survey—hospital patients	SAS
[48]	Israel	03–04.2020	403 pregnant women	Online survey—social media	PRAS
[49]	Italy	03.2020	178 pregnant women	Online survey—hospital patients	STAI
[50]	Italy	03–04.2020	100 pregnant women	Written survey—hospital patients	STAI-SF
[51]	Turkey	04.2020	203 pregnant and 101 non-pregnant women	Written survey—hospital patients	STAI
[52]	USA	04.2020	788 pregnant women	Online survey—social media	GAD-7 PREPS
[53]	USA	04.2020	2740 pregnant women	Online survey—social media	PRAS VAS-anxiety
[54]	USA	04–05.2020	4451 pregnant women	Online survey—social media	PREPS

Abbreviations: Ref—reference; EPDS—Edinburgh Postnatal Depression Scale, GAD-7—Generalized Anxiety Disorder 7; K10—Kessler Distress Scale; PROMIS—Patient-Reported Outcomes Measurement Information System Anxiety Adult form; PRAQ—Pregnancy-Related Anxiety Questionnaire; SDS—Self-Rating Depression Scale; SAS—Self-Rating Anxiety Scale; PHQ—Patient Health Questionnaire; STAI—State-Trait Anxiety Inventory; BDI—Beck Depression Inventory; BAI—Beck Anxiety Inventory; IDAS-II—Inventory of Depression and Anxiety Symptoms II; HADS—The Hospital Anxiety and Depression Scale; PRAS—Pregnancy-Related Anxiety Scale; PREPS—Pandemic-Related Pregnancy Stress Scale; VAS—Visual Analogue Scale. EPDS cut-offs: ^1^ ≥13; ^2^ >13; ^3^ ≥10; ^4^ ≥9/12; ^5^ ≥15.

Of the selected papers, in 22 the participants were pregnant women, in 5 they were perinatal women (pregnant, postpartum and breastfeeding) and in 3 they were postpartum women. In total, 13 papers assessed anxiety and depression symptoms, 8 assessed depression symptoms and 9 assessed anxiety symptoms. Studies came from the USA (7), China (6), Turkey (5), Italy (4), Canada (2), Israel (2), Belgium (1), Qatar (1), Japan (1) and Hong Kong (1). The channels of communication were the Internet in most studies, 12 studies used a written survey or a face-to-face interview, while 1 study used telephone communication. The studies differed in their method of recruitment—most online surveys were distributed via social media, while other studies recruited hospital patients. The study by Sade et al. [40] was conducted among hospitalized patients with high-risk pregnancy.

In six studies, the control group consisted of patients studied before the pandemic or the announcement of the epidemic, and in the study by Ayaz et al. [33], the same patients completed questionnaires before and during the pandemic. Zhou et al. and Yassa et al. [29,51] compared pregnant and non-pregnant women.

To assess the severity of depressive symptoms both specific—Edinburgh Postnatal Depression Scale (EPDS), as well as generic—Beck Depression Inventory (BDI) and Patient Health Questionnaire (PHQ), tools were used [55]. The EPDS was the most commonly used tool to assess depressive symptoms. Studies used different cut-offs to assess depressive symptoms—from ≥10 to ≥15, as indicated in Table 1. Another frequently used tool was the Patient Health Questionnaire (version 2 or 7). The Generalized Anxiety Disorder Scale (GAD-7) and the State-Trait Anxiety Inventory (STAI) were the most commonly used tools to assess anxiety symptoms. Two studies assessed pandemic-related stress experienced by pregnant women using the new instrument, the Pandemic-Related Pregnancy Stress Scale (PREPS). This questionnaire assesses preparedness stress, perinatal infection stress and positive appraisal [56].

### 3.3. Depression and Anxiety Symptoms

The results of studies assessing the prevalence of depressive and anxiety symptoms in perinatal women can be found in Table 2.

**Table 2:** Prevalence of depression and anxiety.

Reference	Symptoms of Depression	Symptoms of Anxiety	Comparator
**Depression and Anxiety**			
[24]	↑ Pregnancy—25.3%; postpartum 23.6%	↑ 39.4% mild; 13.6% moderate-to-severe anxiety	Estimates in Belgium prior to the pandemic
[25]	↑	↑	Pre-pandemic cohort
[26]	↑ 37%	↑ 57%	Similar pre-pandemic pregnancy cohorts
[28]	↑ 50.6%	**↔** 8.3%	Literature data
[29]	↓ 5.3%	**↓** 6.8%	Non-pregnant women during pandemic
[30]	19.2%	31.2%	None
[31]	↑ 34.2% (pregnant women) ↑ 26.3% (postpartum women)	↑ 64% (pregnant women) ↑ 57.7% (postpartum women)	Literature data
[32]	35.4%		None
[33]	↑	↑	Test–retest study
[34]	56.3%	64.5%	None
[35]	12%	60%	None
[36]	9.9%	11.1%	None
[37]	↑ 39.2%	↑ 34.4%	Literature data
**Depression**			
[38]	↑ 29.6%		Pre-alert group
[39]	↑ 14.4%		Pre-alert group
[40]	**↔** 25.0%		Pre-pandemic hospitalized high-risk pregnancy group
[41]	↑ 28.6%		Pre-pandemic control group
[42]	17%		None
[43]	14.7%		None
[44]	**↓** score ≥ 9 in EPDS—15.1% score ≥ 12 8.2%		Pre-restriction group
[45]	24%		None
**Anxiety**			
[46]		17.2%	None
[47]		↑ 14.3%	General population prior to COVID-19
[49]		↑ 77.0%	Literature data
[50]		68%	None
[51]		↑ 62.6%	Literature data
[52]		Mild—35.6%; moderate—21.6%; severe anxiety symptoms—21.7%	None

Meaning of the symbols: ↑—higher than the comparator, ↓—lower than the comparator, **↔** similar to the comparator.

Most studies have shown an increase in the prevalence of depressive symptoms and/or anxiety. Berthelot et al. [25] showed a significant increase in the prevalence of depressive and anxiety symptoms in a group of pregnant patients before and during the COVID-19 pandemic. Similar results were shown by Ayaz et al. [33], who compared the same group of pregnant patients before and during the pandemic. Results of Wu et al. [38] and Hui et al. [39] pointed to an increase in depressive symptoms in perinatal women following the alert announcement regarding coronavirus infection. Zanardo et al. [41] showed an increase in the number of depressed pregnant women during the pandemic compared to a pre-pandemic group in Italy.

On the other hand, the results of Dong et al. [28] showed that, compared to the literature data prior to pandemic, the anxiety level of pregnant women was the same, while the level of depression was significantly higher. In research of Zhou et al. [29], the prevalence of depression and anxiety was lower among pregnant than non-pregnant women. This is partially consistent with the research of Yassa et al. [51] in which both pregnant and non-pregnant women expressed a higher state of anxiety during the pandemic than the normal population in the literature, but the state of anxiety scores were significantly higher in non-pregnant women. In the study by Sade et al. [40] on women hospitalized at the high-risk pregnancy unit during the COVID-19 strict isolation period, the prevalence of depression was high (25.0%), but not higher when compared to women hospitalized before the outbreak of disease.

One study was conducted among pregnant women living in low socioeconomic status environments in New York City [44]. No differences were observed in the mean EPDS values of the women studied in the period before and after the introduction of the restrictions (12 March 2020), but a comparison of the results obtained in the period of 2 February–11 March and in the period of 4 May–12 June indicated decreased symptomatology during restrictions.

### 3.4. Risk Factors

Factors which were connected with a higher risk of depression and/or anxiety are listed in Table 3.

**Table 3:** Risk factors of depression and/or anxiety symptoms.

Perinatal care	Uncertainty and concerns about perinatal care [26,35] Alterations to prenatal appointments [53,54,57] Discomfort with hospital and ambulatory visits [34]
Social factors	Social isolation [26] Lack of social support [31,35,42] Being single [36,42] Partner’s absence at delivery [31] Tension/conflict at home [26,53]
Demographic	Being a woman of color [36,54] Being an Arab woman [48] Education level (high—[49], low—[34,53]) Younger age [36,42]
Financial	Low income, financial difficulties [25,42] COVID-19-related financial stress and income loss [45,54] Unemployment [34,42]
Factors concerning COVID-19	Stress of getting infected with COVID-19 [26,35,42,48,52,53] Suffering subjective symptoms of suspected infection [46] Perceived risk of having had COVID-19 [54] Having infected friends/families/colleagues [57] Self or family member being an essential worker [53] Living in a location with a large number of COVID-19 cases [46,53]
Health state	High-risk pregnancy [48,52,54] Chronic illness [54] Previous psychiatric diagnosis [25,31] Previous adverse experiences during pregnancy [57]
Insufficient information	No information about the effects of COVID-19 [34] Inconsistent messaging from information sources [35]

Studies have offered various factors connected with increased risk of perinatal depression and anxiety. As it is shown in Table 3, women’s worries were related to changes in regular perinatal care and lack of social contacts and support. The lack of presence and support from one’s partner during labor and delivery as well as in the first days postpartum has been found to increase symptoms of anxiety and depression in perinatal women [31].

Higher symptoms of depression and anxiety were associated with more concern about threats of COVID-19 to the life of the mother and baby. In the research of Gur et al. [36], women with COVID-19 and pregnancy worries were more likely to meet the screening threshold for anxiety; only pregnancy-specific worries predicted depression screening results.

Living in a location with a large number of COVID-19 cases has been a significant driver of greater changes in pregnancy-related anxiety scores [53]. In the research of Liu et al. [46], pregnant women in Wuhan were about twice as likely to develop anxiety as women in a less-affected Chinese city; however, this was not confirmed by Dong et al. [28].

Some of the studies we analyzed showed significant demographic, cultural and social differences. A higher risk of depression and/or anxiety was found among unemployed and low-income women [25,34,42]. COVID-19-related financial stress has been significantly associated with increased likelihood of a clinically significant depression score, even after adjustment for covariates including participant education and income [45].

The Taubman–Ben-Ari et al.’s study, which compared Jewish and Arab pregnant women, showed that Arab women reported higher levels of COVID-19-related childbirth anxiety and global fear of childbirth [48]. In research of Gur et al. [36], black women were found to be more likely to meet the criteria for depression than white women, but this difference was not significant when accounting for covariates. Women of color reported higher pandemic-related stress in a study by Preis [54].

### 3.5. Protective Factors

Factors connected with a reduced risk of depression and/or anxiety symptoms are presented in Table 4.

**Table 4:** Protective factors.

Social	Social support [26,47,48] Partner emotional support [35] Low hostility level in close relationships [36] Use of virtual communication platforms [35]
COVID-related information	Information from healthcare workers and televised pandemic-related information [37] More knowledge about COVID-19 [46] Rational perception of COVID-related risk [46,47]
Activity	Physical activity [26,34] Access to outdoor space [35,54] Engagement in various healthy behaviors [35,54]
Personal	More self-reliance [36] Better emotion regulation [36] Positive attitudes towards online medical consultation [46]

Many studies have analyzed factors connected with reduced risk of depression and/or anxiety. In qualitive data gathered by Farewell et al. [35], participants identified various sources of resilience, including the use of virtual communication platforms, engaging in self-care behaviors (e.g., adequate sleep, physical activity and healthy eating), partner emotional support and being outdoors. Lebel et al. [26] found that higher levels of perceived social support and support effectiveness, as well as more physical activity, were associated with lower psychological symptoms. Those with relatively more knowledge about COVID-19 and rational risk perception (not too nervous about epidemic control or going out), were less likely to be anxious [46].

## 4. Discussion

The effects of the COVID-19 pandemic on mental health are still not fully known. Research into this impact in the population and its individual sections is required. It is important to identify which groups are most affected and which methods are most effective in preventing and treating mental disorders in a pandemic [58]. Among the vulnerable groups that need special attention are frontline healthcare workers, children, older people, their caregivers, psychiatric patients and marginalized communities [59]. Pregnant women may also constitute a population vulnerable to mental health disorders during the COVID-19 pandemic. Buekens et al. published a call for action for COVID-19 surveillance and research during pregnancy [10]. Additionally, Caparros-Gonzalez et al. [60] postulated the need to assess symptoms of depression and anxiety in pregnant and postpartum women, as well as the risk and protective factors for these symptoms. This literature review responds, so to speak, to these calls [10]. The studies on mental health of pregnant women and new mothers during COVID-19 pandemic were a response to a changing situation, were conducted in different settings, with different groups of respondents and using different tools. The heterogeneity of study designs and inconsistent results clearly indicate the need for summative research—namely reviews, which contribute to delineate the impact of the pandemic on the mental state of pregnant women and new mothers. Previous systematic reviews [16,17,19,20] and a recent rapid review [18] have focused on the prevalence of anxiety and depression among pregnant women and new mothers.

Our review analyzes a greater number of studies than previous systematic literature reviews and provides a multidimensional examination of the prevalence of depressive and anxiety symptoms in perinatal women, including risk and protective factors, which can contribute to the development of recommendations for healthcare professionals. Our multidisciplinary research group includes academics and practitioners, doctors from different specialties, psychologists, psychotherapists and practicing midwives. We applied the integrative review method that allows for the combination of diverse methodologies to provide a comprehensive understanding of a particular phenomenon or healthcare problem [21] and has the potential to be directly applied to practice and policy.

### 4.1. Anxiety and Depression Symptoms in Pregnant Women and New Mothers

The studies included in our review showed a great deal of diversity. To minimize this factor as much as possible, we limited the review to papers on the first wave of COVID-19—between March and June 2020 [61,62]. We included only papers using validated tools and we focused only on symptoms of anxiety and depression; for clarity reasons, we did not analyze other psychological problems such as insomnia and post-traumatic stress disorder symptoms. The diversity of the papers analyzed also has positive aspects, as it highlights the importance of different risk and protective factors, which can be used in the development of interventions.

The results of our literature review indicate a significant prevalence of depressive and anxiety symptoms in perinatal women during the COVID-19 pandemic. Most of the studies analyzed involved pregnant women [25,26,27,28,29,32,33,34,36,38,40,42,44,45,46,47,48,49,50,51,52,53,54], five involved a mixed group of pregnant and postpartum women [24,30,31,35,37] and three studies involved new mothers [39,41,43]. Significant differences in the prevalence of depressive symptoms (5.3% to 56.3%) and anxiety (6.8% to 77%) in the studied population were noted. This is due to the aforementioned heterogeneity of the study groups, the different somatic status of women (high-risk pregnancy vs. normal pregnancy), the situation of the study subjects (at home vs. in hospital), the different economic status of the study subjects (some studies were conducted in a group of women with low socioeconomic status) and ethnicity (women of color, white women, Arab and Jewish women). In most of the studies we observed an increase in depressive and/or anxiety symptoms during the pandemic period [7,24,25,26,28,31,33,37,38,39,47,49].

### 4.2. Risk and Protective Factors

The pandemic has significantly changed the lives of expectant and new mothers, reducing their sense of security, limiting social support and causing social isolation. Some of the risk factors identified in our study are closely related to the pandemic, such as fear of infecting oneself, the child and loved ones. Pregnant women are among those who have higher levels of stress related to worries about getting infected or spreading COVID-19 [9]. The women interviewed also pointed to the importance of changes in the organization of perinatal care, online visits and not having a partner at the birth. However, it should be remembered that typical risk factors for mental disorders in the perinatal period also increase in importance during the pandemic (e.g., chronic diseases, low income). Analysis of the studies also made it possible to identify protective factors reducing depression and anxiety symptoms. Social support, especially a partner’s emotional support and the woman’s own activity during the perinatal period (physical activity, engaging in self-care behaviors) play a significant role here. Several studies have highlighted the role of knowledge about COVID-19 [26,35,42,48,53] and the importance of information provided by healthcare workers [37].

### 4.3. Practical Implications

In the fight against the COVID-19 pandemic, policymakers and healthcare professionals focus primarily on preventing the spread of the virus and treating those infected. The results of our review made it possible to identify the mental health needs of pregnant women and new mothers and to formulate practical recommendations for action to be taken in primary healthcare and in specialist gynecological-obstetric care. We divided the recommendations into three areas of action: information, screening and intervention.

#### 4.3.1. Information for Pregnant Women and New Mothers

Information for pregnant women and new mothers should address the following issues:

- Uncertainty about COVID-19 and about the impact it may have on pregnancy and perinatal health. This information should come from authoritative sources [46] and be evidence based [34,35];
- Recommendations concerning social isolation behaviors, breastfeeding and impacts of disasters on mental and physical health [35];
- Infection prevention procedures for labor, delivery and postpartum [52];
- The way medical and midwife care is provided in the time of COVID-19 and methods of conducting visits (in the surgery and online) [35];
- Information about mental health and ways to relieve stress among pregnant women and encouraging the use of reliable sources [25,46];
- Information on how to get help and/or psychological support [35].

Information should be provided as early as possible: obtaining it can alleviate some stress and subsequent anxiety [52]. Different ways of providing information should be used, including information technology (websites of the government, ministry of health, healthcare facilities), toll-free helplines answering questions of pregnant women, handouts and information provided by healthcare professionals during visits both in the surgery and online, as well as when arranging appointments. It has been demonstrated that women who had more knowledge about COVID-19 provided by medical professionals showed lower levels of anxiety and depressive symptoms [34]. Attention should therefore be paid to women, particularly by providing information to those who are younger, affiliated to the subsidized regime and with lower levels of education, as this group usually has less knowledge about COVID-19 [63].

#### 4.3.2. Assessment of Risk Factors and Screening for Depression/Anxiety Symptoms

- Mental health issues should be addressed during visits—questions should be asked about perceived stress, symptoms of mental health problems and social support [25];
- Screening for psychosocial vulnerabilities and depression/anxiety symptoms should be performed during healthcare visits in all pregnant and postpartum women [35,52];
- It is necessary to pay particular attention to patients from the depression or anxiety risk groups in the times of COVID-19, i.e., single and without social support, younger age, with financial difficulties, unemployed, those with high stress of getting infected, with high-risk pregnancy, with chronic illness, with previous psychiatric diagnosis and with previous adverse experiences during pregnancy;
- It is important to continue to monitor the mood of postpartum patients, especially women with anxiety and depressive disorders in pregnancy [53];
- Remote questionnaires may prove useful in identifying women with mental health problems [49].

#### 4.3.3. Interventions in Women with Perinatal Mental Health Problems

In this group of patients, collaboration between perinatal care and mental health services is essential. It is crucial to recognize when consultation/treatment by mental health professionals is necessary. As mentioned above, in perinatal care it should be compulsory for every pregnant woman to be assessed for mental health issues at every stage of her pregnancy and postpartum [64]. Standardized tools can be useful here [55], and questionnaires can also be used remotely [49]. Mental status assessments conducted in perinatal healthcare settings includes suicide risk assessment. In psychiatric settings, suicide risk assessment is one component of an investigation conducted to increase patient safety [65]; asking about suicidal thoughts of a patient who has come to a psychiatrist is considered appropriate. However, healthcare professionals working outside the mental healthcare setting believe that these questions could induce thoughts of self-harm and suicide risk [66], and often avoid asking them. The results of the review by Dazzi et al. [67] suggest that acknowledging and talking about suicide may in fact reduce, rather than increase, suicidal ideation, and may lead to improvements in mental health in treatment-seeking populations. Women with mental health issues should be offered psychiatric consultations and psychological support; sometimes urgent psychiatric hospitalization is necessary. The treatment depends on the condition of the patient taking into account the risk of suicide. Some patients require psychological support and counseling [25,34,46], and during the pandemic period, therapy often takes place online.

As mentioned above, telehealth options may be used in screening, treating and monitoring perinatal mental health [53]. The COVID-19 pandemic forced the prenatal care system to reorganize efforts toward more virtual visits [68]. Telehealth in psychiatric care was already developing before the COVID-19 pandemic, and the current situation has confirmed its usefulness in prenatal care [25,53,69,70,71]. Virtual visits make it possible to provide social distance while reaching people in need quickly, allowing for screening, monitoring and therapy of mental disorders.

Obstetricians, midwives and other health professionals in perinatal care play a crucial role in ensuring health interventions for women nearing or after childbirth. It is more important than ever to carry out activities to promote awareness among perinatal women and identify symptoms of depression and anxiety. Healthcare professionals can be involved in mental health promotions and interventions in pregnant women by planning physical activity for them and encouraging them to engage in social activities [34]. Brief interventions to improve self-efficacy and motivate women to engage in healthy activities could be useful in decreasing anxiety [40,52].

### 4.4. Limitations

Our study has a number of limitations. First, as mentioned previously, the comparability of study results was complicated by a diversity of definitions and measurements of perinatal depression and anxiety. Second, we only included studies conducted during the first wave of COVID-19. Third, a significant proportion of the studies in our review used the Internet for data collection. Although a growing number of researchers are relying on this method, data collected in this way may include better educated, more technology-savvy respondents and exclude perinatal women who do not have access to a computer and the Internet; these minority populations are more impacted by COVID-19 and underrepresented in the studied group [53]. Limitations at the review level have to be stressed. The protocol was not registered prior to the study and only two databases were searched. The eligibility criteria included only articles published in English-language journals. Whereas the screening was performed independently by two reviewers, the level of agreement concerning eligibility was not calculated. A meta-analysis of findings was not performed.

### 4.5. Long-Term Effects of the COVID-19 Pandemic

The COVID-19 pandemic prompted the identification of priorities and long-term research strategies for mental health [72]. Lessons from previous pandemics have helped develop guidelines for international public health strategies [73]. In the context of care for pregnant women and newborn babies, the Center for Disease Control and Prevention developed interim guidelines for obstetric care [74]. However, these recommendations relate to somatic health management. Our findings support the postulate of Thapa et al. [75] and Holmes et al. [72] that appropriate management strategies for the mental health of pregnant women should be developed. Health needs of the population during a pandemic are considered as four ‘waves’ [76,77] of the pandemic. The first wave involves the immediate mortality and morbidity of COVID-19 pandemic. In many countries most of the efforts of policymakers and healthcare workers are directed towards this first wave. However, it is necessary to simultaneously mitigate the other waves and plan further actions directed at them [77]. The second wave is due to the fact that patients with acute conditions (e.g., myocardial infarction) do not receive care because of lack of access to a specialist or the fact that they do not see a doctor for fear of infection. The third wave is related to the lack of routine care for patients with chronic diseases (also due to patients’ fear of infection, service reduction). These patients include people with diabetes or mental disorders who are in therapy and require regular visits; as a result of a break in treatment, their condition deteriorates. The fourth wave of healthcare needs is the largest and will last the longest, due to the psychosocial and mental burden of the pandemic. It will likely come to the fore when the spread of the virus is under control. We speculate that the fourth wave will last months or even years after the end of the pandemic. Many of the mental health needs of pregnant and postpartum women are met in primary care or perinatal care services, but some require specialist interventions in mental health settings. Further interventions are needed to prepare for and reduce the severity of the fourth wave [77]. In women at risk of depression counseling, approaches such as interpersonal therapy and cognitive behavioral therapy can be effective [78]. Social support and stress-reduction interventions to promote maternal mental health should be also implemented [79]. 

It should be stressed that even before the COVID-19 pandemic, perinatal mental health problems were underdiagnosed. Data suggest that only 30%–50% of women with perinatal depression were identified in clinical settings and approximately 15% of them received treatment [80]. The results of our review indicate that, in most of the studies conducted so far during pandemic, an increase in the prevalence of depression and/or anxiety in perinatal women has been observed. This may only be the beginning, as the social and psychologic effects of the pandemic continue [81]. Our study identifies protective and risk factors and provides recommendations concerning the management of mental health problems in perinatal healthcare settings. It may therefore contribute to the development of strategies for protecting maternal mental health during and after the COVID-19 pandemic.

## Data Availability

Not applicable.
